# Spectrum and antimicrobial resistance in acute exacerbation of chronic obstructive pulmonary disease with pneumonia: a cross-sectional prospective study from Vietnam

**DOI:** 10.1186/s12879-024-09515-6

**Published:** 2024-06-23

**Authors:** Duy Tuyen Dao, Huu Y Le, Minh Hai Nguyen, Thi Duyen Thi, Xuan Dung Nguyen, Thanh Thuyet Bui, Thi Huyen Trang Tran, Van Luan Pham, Hang Nga Do, Jim-Tong Horng, Huu Song Le, Dinh Tien Nguyen

**Affiliations:** 1 Department of Respiratory Diseases, 108 Military Central Hospital, Hanoi, Vietnam; 2 Graduate Institute of Biomedical Sciences, College of Medicine, Chang Gung University, Taoyuan, Taiwan; 3 Faculty of Respiratory Medicine, 108 Institute of Clinical Medical and Pharmaceutical Sciences, Hanoi, Vietnam; 4 Vietnamese-German Center for Medical Research (VG-CARE), 108 Military Central Hospital, Hanoi, Vietnam; 5 Department of Microbiology, 108 Military Central Hospital, Hanoi, Vietnam; 6 Department of Molecular Biology, 108 Military Central Hospital, Hanoi, Vietnam; 7 Department of Biochemistry and Molecular Biology, College of Medicine, Chang Gung University, Taoyuan, Taiwan; 8 Research Center for Emerging Viral Infections, College of Medicine, Chang Gung University, Taoyuan, Taiwan; 9 Molecular Infectious Disease Research Center, Chang Gung Memorial Hospital, Chang Gung University College of Medicine, Taoyuan, Taiwan; 10 Research Center for Industry of Human Ecology and Research Center for Chinese Herbal Medicine, Graduate Institute of Health Industry Technology, Chang Gung University of Science and Technology, Taoyuan, Taiwan; 11 Faculty of Infectious Diseases, 108 Institute of Clinical Medical and Pharmaceutical Sciences, Hanoi, Vietnam

**Keywords:** Acute exacerbation, Antibiotic resistance, Bacteriology, Chronic obstructive pulmonary disease, Pneumonia

## Abstract

**Background:**

Respiratory infections have long been recognized as a primary cause of acute exacerbation of chronic obstructive pulmonary disease (AE-COPD). Additionally, the emergence of antimicrobial resistance has led to an urgent and critical situation in developing countries, including Vietnam. This study aimed to investigate the distribution and antimicrobial resistance of bacteria in patients with AE-COPD using both conventional culture and multiplex real-time PCR. Additionally, associations between clinical characteristics and indicators of pneumonia in these patients were examined.

**Methods:**

This cross-sectional prospective study included 92 AE-COPD patients with pneumonia and 46 without pneumonia. Sputum specimens were cultured and examined for bacterial identification, and antimicrobial susceptibility was determined for each isolate. Multiplex real-time PCR was also performed to detect ten bacteria and seven viruses.

**Results:**

The detection rates of pathogens in AE-COPD patients with pneumonia were 92.39%, compared to 86.96% in those without pneumonia. A total of 26 pathogenic species were identified, showing no significant difference in distribution between the two groups. The predominant bacteria included *Klebsiella pneumoniae, Haemophilus influenzae, Moraxella catarrhalis*, and *Streptococcus pneumoniae*, followed by *Acinetobacter baumannii* and *Streptococcus mitis*. There was a slight difference in antibiotic resistance between bacteria isolated from two groups. The frequency of *H. influenzae* was notably greater in AE-COPD patients who experienced respiratory failure (21.92%) than in those who did not (9.23%). *S. pneumoniae* was more common in patients with stage I (44.44%) or IV (36.36%) COPD than in patients with stage II (17.39%) or III (9.72%) disease. ROC curve analysis revealed that C-reactive protein (CRP) levels could distinguish patients with AE-COPD with and without pneumonia (AUC = 0.78).

**Conclusion:**

Gram-negative bacteria still play a key role in the etiology of AE-COPD patients, regardless of the presence of pneumonia. This study provides updated evidence for the epidemiology of AE-COPD pathogens and the appropriate selection of antimicrobial agents in Vietnam.

**Supplementary Information:**

The online version contains supplementary material available at 10.1186/s12879-024-09515-6.

## Background

Chronic obstructive pulmonary disease (COPD) is a progressive lung disease characterized by airflow restriction and persistent respiratory symptoms [1]. It is one of the leading causes of morbidity and mortality worldwide, accounting for more than 3.5 million deaths each year, with nearly 90% of deaths occurring in low- and middle-income countries. Exacerbations significantly contribute to this phenomenon [1]. In Vietnam, the prevalence of COPD is estimated to range from 7 to 10% [2–4]. Notably, more than half of COPD patients experience exacerbations, leading to hospitalization and increasing the economic burden on both individuals and the health system [3].

Acute exacerbations of COPD (AE-COPD) are defined as acute worsening of respiratory symptoms that result in additional therapy characterized by increased dyspnea and/or cough and sputum [5]. The cause of AE-COPD may be multifactorial and include respiratory tract infections (either viral or bacterial) and noninfectious factors (such as air pollution, meteorological effects, and comorbidities) [6]. Among these, bacterial infection is considered a primary risk factor for infective AE-COPD, the prevalence of which ranges from 26 to 81% [6]. The dominant bacteria isolated from the patients with AE-COPD were *S. pneumoniae, H. influenzae, M. catarrhalis, A. baumannii, P. aeruginosa*, and *S. aureus* [7]. In several studies, the presence of bacteria in AE-COPD patients has been correlated with sputum purulence and the presence of inflammatory markers associated with pneumonia [8, 9]. Pneumonia in AE-COPD remains controversial. However, it is noted that pneumonia frequently occurs in hospitalized COPD patients and has the potential to worsen the progression of COPD exacerbations [10]. It is often not recognized and tends to be underdiagnosed due to the similarity of symptoms to those of AE-COPD. This has implications for accurately stratifying risk and providing appropriate treatment for COPD patients [11]. Additionally, there is limited knowledge regarding the clinical characteristics and pathogenic differences between AE-COPD patients with and without pneumonia [12, 13]. Conducting a comprehensive study on this matter could significantly enhance our understanding and refine treatment strategies for these patients.

Traditional techniques, such as sputum culture, primarily identify bacteria and their antibiotic susceptibilities. However, they are limited to detect viruses and atypical bacteria effectively [14]. In contrast, real-time PCR demonstrates notable efficacy in identifying respiratory viruses and a broader spectrum of bacterial strains, both typical and atypical. Nevertheless, its capability to solely detect antibiotic resistance genes [15], rather than complete resistance profiles, poses a limitation. Therefore, a combined approach utilizing both methodologies is advisable for comprehensive respiratory pathogen detection and antibiotic resistance assessment. Currently, more than 90% of AE-COPD patients receive antibiotics as the primary treatment, depending on the severity of both COPD and the exacerbation, irrespective of the presence or absence of pneumonia [8, 16, 17]. However, the efficacy of many of these drugs has become uncertain due to the emergence of resistant strains, which are the most common respiratory pathogens in different countries [16]. The selection of antibiotics is heavily influenced by the local prevalence of bacteria and their resistance [18]. Therefore, in addition to improving antimicrobial restriction systems [19], understanding this issue is essential for preventing disease progression and refining treatment protocols for the effective management of AE-COPD. To our knowledge, there is limited information available in the literature regarding the epidemiology and antibiotic susceptibility between AE-COPD patients with and without pneumonia.

For the reasons mentioned above, our study aimed to determine the pathogenic distribution of COPD exacerbation between these two groups using both conventional culture and multiplex real-time PCR. We further analyzed the antimicrobial sensitivity of the isolates and their associations with several clinical characteristics as well as indicators of pneumonia among hospitalized AE-COPD patients at 108 Military Central Hospital, one of the largest hospitals in Vietnam.

## Materials and methods

### Patients and study design

This study was conducted among patients with AE-COPD admitted to 108 Military Central Hospital in Hanoi, Vietnam, due to acute respiratory illness. All participants underwent clinical examinations, laboratory tests, spirometry, and chest computed tomography (CT) within 24 h of admission. The diagnosis of COPD or AE-COPD, along with COPD-related evaluations in this study, followed the guidelines of the Global Initiative for Chronic Obstructive Lung Disease (GOLD) [5]. Pneumonia was defined according to the National Institute for Health and Care Excellence (NICE) guidelines [20] and a prior study [11]. We excluded patients identified with an alternative cause at admission and those with immunosuppression. A total of 92 AE-COPD patients with pneumonia and 46 without pneumonia (as controls) were ultimately recruited from September 2020 to September 2023 for this cross-sectional prospective study.

### Bacterial culture and identification

Sputum was collected and promptly processed within a maximum of 4 h if stored at 4 °C. Samples with fewer than 10 epithelial cells and/or 25 polymorphonuclear leukocytes per low-power field (×100 microscopy) were qualified and subsequently plated onto agar plates (blood agar, chocolate agar, and MacConkey agar) for culture. After 18–24 h of incubation at 37 °C under 5% CO_2_, the bacterial species were identified via colony characteristics and biochemical reactions using a Vitek® 2 compact system (bioMérieux, France) with GP ID cards. Isolates of the same species from one patient were considered to be a single isolate. The entire process strictly adhered to protocols described in the *Manual of Clinical Microbiology* [21].

### Multiplex real-time PCR for bacterial and viral detection

Sputum samples were first homogenized with a mucolytic reagent (N-acetyl-L-cysteine), and nucleic acid was subsequently extracted using the SaMag nucleic acid extraction kit (Sacace, Italy) following the manufacturer’s protocol. The quality of samples was evaluated by measuring the optical density (OD) at 260 nm for DNA and 230 nm for RNA using a NanoDrop™ Spectrophotometer (Thermo Fisher Scientific, Inc., MA, USA). Multiplex one-step real-time RT-PCR was performed on the CFX96™ (Bio-Rad, CA, USA) platform using the Allplex™ Respiratory Panel 1, 2, 3, and 4 kits (Seegene, Korea) according to their instructions and previous studies [22, 23] for the detection of *Streptococcus pneumoniae, Haemophilus influenzae, Mycoplasma pneumoniae, Chlamydia pneumoniae*, *Legionella pneumophila*, and seven viruses, including *influenza A/B virus, respiratory syncytial virus A/B, adenovirus, enterovirus, parainfluenza virus, rhinovirus, and coronavirus*. In addition, an in-house multiplex real-time RT-PCR assay was used to identify common bacterial pathogens, including *Klebsiella pneumoniae, Moraxella catarrhalis, Staphylococcus aureus, Pseudomonas aeruginosa*, and *Acinetobacter baumannii* following the standard methods in previous report [24].

### Antimicrobial susceptibility testing

The positive samples with bacterial culture were further tested for antimicrobial susceptibility according to CLSI guidelines [25] and a prior study [26]. The bacterial colonies were first resuspended at turbidity levels specific to various microorganisms. The antibiotic susceptibility of these samples was subsequently tested using a Vitek® 2 compact system (bioMérieux, France) with appropriate ID cards according to the manufacturer’s instructions. The selection of antibiotic groups was based on the bacterial species and clinical relevance. The minimum inhibitory concentration (MIC) was automatically recorded and analyzed. MIC values were classified as sensitive (S), intermediate (I) or resistant (R).

### Statistical analysis

Continuous variables are presented as the mean ± standard deviation (SD), while categorical variables are expressed as numbers and percentages. The comparison of characteristics between two groups was conducted by using a *t* test for continuous variables and a χ^2^ test and Fisher’s exact test for categorical variables. To determine whether biomarkers could distinguish AE-COPD patients with and without pneumonia, receiver operating characteristic (ROC) analysis and area under the curve (AUC) calculation were performed. The significance level was set at a *p* value less than 0.05. All the data were statistically analyzed using SPSS version 25.0 (NY, USA).

## Results

### Baseline characteristics

The baseline characteristics of 92 AE-COPD patients with pneumonia and 46 controls are reported in Table 1. Among them, there were 77 (83.70%) and 43 (93.48) males, and the mean ± SD ages at admission were 76.86 ± 10.24 and 74.65 ± 9.28 years, respectively. There were no differences in the duration of illness, history of inhaled corticosteroid (ICS) use, and group and stage classification of COPD between the two groups. The incidence of fever and respiratory failure was significantly greater in AE-COPD patients with pneumonia than controls (*p* < 0.01). The AE-COPD with pneumonia also had higher Modified Medical Research Council (mMRC) dyspnea and COPD Assessment Test (CAT) scores (*p* < 0.01). We also investigated the severity of AE-COPD, and the results showed that the severe grade in the pneumonia group (65.22%) was significantly higher than that in AE-COPD only group (19.57%), *p* < 0.001. Similar phenomena were observed for the total white blood cell (WBC) count, neutrophil percentage, and C-reactive protein (CRP) and procalcitonin (PCT) levels, but not for eosinophil count, with *p* < 0.05.

**Table 1:** Characteristics of AE-COPD patients with and without pneumonia

Characteristics		AE-COPD with pneumonia (*N* = 92)	AE-COPD only (*N* = 46)	*p*
Age (year)		76.86 ± 10.24	74.65 ± 9.28	0.221
Male		77 (83.70)	43 (93.48)	0.10
Duration of illness (year)		7.23 ± 4.38	7.21 ± 5.62	0.980
History of ICS use		50 (54.35)	27 (58.70)	0.14
Fever		53 (57.61)	10 (21.74)	0.001
Respiratory failure		56 (60.87)	17 (36.96)	0.008
mMRC		3.11 ± 0,50	2.74 ± 0,61	0.001
CAT		22.27 ± 3.14	19.70 ± 3.16	< 0.001
Group of COPD	B	7 (7.61)	6 (13.04)	0.30
	D	85 (92.39)	40 (86.96)	
Stage of COPD	I	6 (6.52)	3 (6.52)	0.056
	II	36 (39.13)	10 (21.74)	
	III	46 (50.00)	26 (56.52)	
	IV	4 (4.35)	7 (15.22)	
Severity of AE-COPD	Nonsevere	32 (34.78)	37 (80.43)	< 0.001
	Severe	60 (65.22)	9 (19.57)	
WBC (µL^-1^)		14,000 ± 666	10,610 ± 469	0.001
Neutrophils (%)		80.41 ± 11.07	71.44 ± 15.75	0.001
Eosinophils (µL^− 1^)		157.48 ± 258.17	429.98 ± 845.31	0.043
CRP (mg/L)		82.91 ± 85.11	20.12 ± 26.58	< 0.001
PCT (ng/mL)		1.83 ± 6.38	0,40 ± 0.96	0.039

Values are given as the mean ± SD or number (%)

AE-COPD, acute exacerbation of chronic obstructive pulmonary disease; ICS, inhaled corticosteroid; mMRC, Modified Medical Research Council; CAT, COPD Assessment Test; WBC, white blood cell; CRP, C-reactive protein; PCT, procalcitonin

### Comparison of pathogen distribution between AE-COPD patients with and without pneumonia

A thorough investigation of pathogen identification was conducted utilizing real-time RT-PCR and bacterial culture in AE-COPD patients in both groups. As shown in Table 2, real-time PCR detected pathogens in pneumonia and control groups at rates of 79.35% and 67.39%, respectively. Bacterial culture identified at rates of 54.35% and 65.22%, respectively. Overall, employing dual techniques in tandem resulted in pathogen detection rates of 92.39% in pneumonia patients and 86.96% in controls. We conducted a detailed analysis of the subgroups of detected pathogens. The majority of patients were positive for a single pathogen, comprising 69.41% (59/85) of patients in the pneumonia group and 75.00% (30/40) of those with only AE-COPD. Notably, single Gram-negative bacteria were the most prevalent with 52.94% (45/85) and 50% (20/40), respectively. However, there were no significant differences observed between these two groups (all *p* > 0.05) (Table 2). We also performed blood cultures for all participants. However, only one sample in each group was positive (Table S1).

**Table 2:** Pathogens detected in AE-COPD patients with and without pneumonia

Pathogens	Multiplex real-time PCR		Bacterial culture		Total		
	AE-COPD with pneumonia (*N* = 92)	AE-COPD only (*N* = 46)	AE-COPD with pneumonia (*N* = 92)	AE-COPD only (*N* = 46)	AE-COPD with pneumonia (*N* = 92)	AE-COPD only (*N* = 46)	*p*
**Identified results**							
Pathogen detected	73 (79.35)	31 (67.39)	50 (54.35)	30 (65.22)	85 (92.39)	40 (86.96)	0.30
No pathogen detected	19 (20.65)	15 (32.61)	42 (45.65)	16 (34.78)	7 (7.61)	6 (13.04)	
**Number of pathogen detected**							
Single	47 (64.38)	21 (67.74)	50 (100)	30 (100)	59 (69.41)	30 (75.00)	0.41
Mixed	26 (35.62)	10 (32.26)	0 (0)	0 (0)	26 (30.59)	10 (25.00)	
**Pathogen detected subgroups**							
Single Gram-negative bacteria	42 (57.53)	17 (54.84)	40 (80.00)	24 (80.00)	45 (52.94)	20 (50.00)	0.31
Single Gram-positive bacteria	3 (4.11)	3 (9.68)	10 (20.00)	6 (20.00)	12 (14.12)	9 (22.50)	
Virus only	2 (2.74)	1 (3.22)	ND	ND	2 (2.35)	1 (2.50)	
Mixed bacteria	23 (31.51)	8 (25.81)	0 (0)	0 (0)	23 (27.06)	8 (20.00)	
Mixed bacteria and virus	3 (4.11)	2 (6.45)	ND	ND	3 (3.53)	2 (5.00)	

The values are given as numbers (%)

AE-COPD, acute exacerbation of chronic obstructive pulmonary disease; ND, not determined

Table 3 provides a comprehensive overview of the pathogen distribution detected by each method employed. Notably, according to both multiplex real-time PCR and bacterial culture analyses, *K. pneumoniae*, a Gram-negative bacterium, emerged as the most prevalent pathogen, accounting for 55.43% and 56.52% of pneumonia patients and controls, respectively. Additionally, other significant Gram-negative bacteria, such as *H. influenzae*, *M. catarrhalis*, and various *Acinetobacter* spp., were identified, even though with varying frequencies across the sampled population. Among the Gram-positive bacteria, *S. pneumoniae* was the predominant species and was detected in 17.39% and 15.22% of the samples within these two groups, respectively. Supplementary Gram-positive species included *S. aureus* and *S. mitis*, albeit in smaller proportions. Atypical bacteria and viruses were also detected. Furthermore, the presence of mixed bacterial and viral infections suggested that multiple infections were prevalent in AE-COPD patients. These findings underscore the importance of employing an integrated approach to yield more precise results and achieve a higher detection rate of pathogens, particularly through multiplex real-time PCR (see Tables 2 and 3 for details).

**Table 3:** Pathogen distribution between AE-COPD patients with and without pneumonia

Pathogens	Multiplex real-time PCR		Bacterial culture		Total	
	AE-COPD with pneumonia (*N* = 92)	AE-COPD only (*N* = 46)	AE-COPD with pneumonia (*N* = 92)	AE-COPD only (*N* = 46)	AE-COPD with pneumonia (*N* = 92)	AE-COPD only (*N* = 46)
**Gram-negative bacteria**						
*Klebsiella pneumoniae*	49 (53.26)	24 (52.17)	10 (10.87)	6 (13.04)	51 (55.43)	26 (56.52)
*Haemophilus influenzae*	18 (19.57)	4 (8.70)	2 (2.17)	0 (0)	18 (19.57)	4 (8.70)
*Moraxella catarrhalis*	7 (7.61)	3 (6.52)	8 (8.70)	9 (19.57)	8 (8.70)	9 (19.57)
*Acinetobacter baumannii*	1 (1.09)	0 (0)	7 (7.60)	3 (6.52)	7 (7.61)	3 (6.52)
*Pseudomonas aeruginosa*	0 (0)	0 (0)	1 (1.09)	1 (2.17)	1 (1.09)	1 (2.17)
*Escherichia coli*	ND	ND	4 (4.35)	0 (0)	4 (4.35)	0 (0)
*Stenotrophonas metophilia*	ND	ND	2 (2.17)	1 (2.17)	2 (2.17)	1 (2.17)
*Enterobacter aerogenes*	ND	ND	1 (1.09)	1 (2.17)	1 (1.09)	1 (2.17)
*Klebsiella oxytoca*	ND	ND	1 (1.09)	0 (0)	1 (1.09)	0 (0)
*Brevundimonas vesicularis*	ND	ND	1 (1.09)	0 (0)	1 (1.09)	0 (0)
*Acinetobacter ursingii*	ND	ND	1 (1.09)	0 (0)	1 (1.09)	0 (0)
*Achromobacter xylosoxidans*	ND	ND	1 (1.09)	0 (0)	1 (1.09)	0 (0)
*Elizabethkingia anophelis*	ND	ND	0 (0)	1 (2.17)	0 (0)	1 (2.17)
*Acinetobacter pittii*	ND	ND	1 (1.09)	0 (0)	1 (1.09)	0 (0)
*Acinetobacter nosocomialis*	ND	ND	0 (0)	1 (2.17)	0 (0)	1 (2.17)
*Acinetobacter junii*	ND	ND	0 (0)	1 (2.17)	0 (0)	1 (2.17)
*Klebsiella oxytoca*	ND	ND	1 (1.09)	0 (0)	1 (1.09)	0 (0)
*Brevundimonas vesicularis*	ND	ND	1 (1.09)	0 (0)	1 (1.09)	0 (0)
**Gram-positive bacteria**						
*Streptococcus pneumoniae*	16 (17.39)	7 (15.22)	2 (2.17)	0 (0)	16 (17.39)	7 (15.22)
*Staphylococcus aureus*	1 (1.09)	0 (0)	1 (1.09)	0 (0)	2 (2.17)	0 (0)
*Streptococcus mitis*	ND	ND	3 (3.26)	5 (10.87)	3 (3.26)	5 (10.87)
*Streptococcus salivarius*	ND	ND	1 (1.09)	1 (2.17)	1 (1.09)	1 (2.17)
*Streptococcus constellatus*	ND	ND	1 (1.09)	0 (0)	1 (1.09)	0 (0)
**Atypical bacteria**						
*Mycoplasma pneumoniae*	3 (3.26)	1 (2.17)	ND	ND	3 (3.26)	1 (2.17)
*Legionella pneumophila*	3 (3.26)	1 (2.17)	ND	ND	3 (3.26)	1 (2.17)
**Virus**						
*Influenza A/B virus*	5 (5.43)	3 (6.52)	ND	ND	5 (5.43)	3 (6.52)

The values are given as numbers (%)

*p* > 0.05 between these two groups

AE-COPD, acute exacerbation of chronic obstructive pulmonary disease; ND, not determined

### Antibiotic susceptibility

**Fig. 1:**
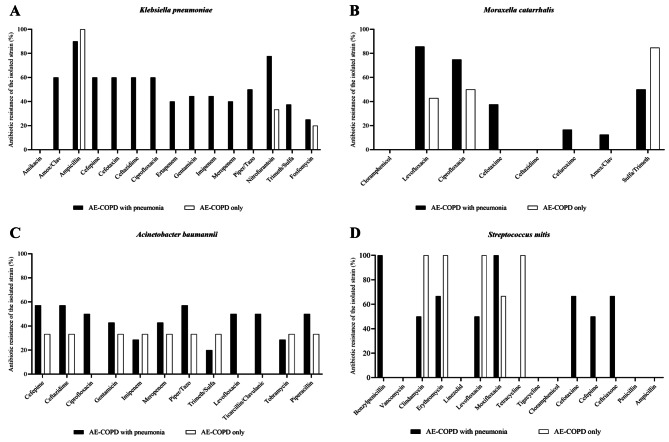
The antibiotic resistance profiles of bacteria isolated from the two groups. The percentage of resistance to standard antibiotic doses was determined for *Klebsiella pneumoniae* (**A**), *Moraxella catarrhalis* (**B**), *Acinetobacter baumannii* (**C**), and *Streptococcus mitis* (**D**). AE-COPD, acute exacerbation of chronic obstructive pulmonary disease

To assess antibiotic resistance patterns and enhance the clarity of treatment strategies, we conducted comprehensive antimicrobial susceptibility testing on bacterial isolates, with a particular focus on the most prevalent pathogenic species. Due to the limited number of positive culture samples for *H. influenzae* and *S. pneumoniae* (Table 3 and S2, S3), the four species included in this assay were *K. pneumoniae*, *M. catarrhalis*, *A. baumannii*, and *S. mitis* (Fig. 1).

We observed a distinct reversal in the antibiogram analysis of *K. pneumoniae*. In the control group, all antibiotics except ampicillin, nitrofurantoin, and fosfomycin exhibited complete sensitivity, reaching up to 100%. However, in the patients with pneumonia, resistance to all antibiotics was evident (Fig. 1A). *M. catarrhalis* demonstrated susceptibility to ceftazidime and chloramphenicol in both groups, with rates exceeding 80%. However, variations in resistance emerged during testing with levofloxacin, ciprofloxacin, and sulfamethoxazole/trimethoprim. Notably, in the AE-COPD with pneumonia patients, the percentage resistant to cefotaxime increased to 50% compared to that in controls, which maintained full sensitivity (Fig. 1B). *A. baumannii*, a Gram-negative bacterium, has become a cause for concern due to its alarming antibiotic resistance to cefepime, ceftazidime, piperacillin, and piperacillin/tazobactam in both cohorts. Interestingly, while several antibiotics, including ciprofloxacin, levofloxacin, and ticarcillin/clavulanic acid, were still effective at treating AE-COPD only patients, their effectiveness significantly decreased to 60% in the presence of pneumonia (Fig. 1C).

Additionally, an antibiogram was generated for *S. mitis*, a Gram-positive bacterium predominantly identified in this study through the sputum culture method ( (Fig. 1D). Our findings revealed that vancomycin, linezolid, tigecycline, chloramphenicol, penicillin, and ampicillin were effective at treating both groups of patients. However, some differences in antibiotic resistance between the two groups were observed. While benzylpenicillin, cefotaxime, cefepime, and ceftriaxone displayed higher resistance in AE-COPD with pneumonia, the susceptibility to clindamycin, erythromycin, levofloxacin, and tetracycline notably decreased in the group with only AE-COPD. These results suggested the nuanced response of bacterial strains to antibiotic treatments, particularly in the context of pneumonia in AE-COPD patients.

### Associations with respiratory failure and stage of COPD

The relationships between several clinical characteristics of AE-COPD patients and the four most common bacteria are shown in Table 4. Among patients positive for *K. pneumoniae* and *M. catarrhalis*, no significant differences were observed in respiratory failure status, stage and group of COPD, and severity of AE-COPD. Interestingly, the frequency of *H. influenzae* infection was notably greater in AE-COPD patients who experienced respiratory failure (21.92%) than in those did not (9.23%), with *p* = 0.042. Furthermore, a higher incidence of *S. pneumoniae* was observed among patients with stage I (44.44%) or IV (36.36%) COPD than among those with stage II (17.39%) or III (9.72%) disease, with a *p* value of 0.014.

**Table 4:** Associations between several clinical characteristics and the top four most prevalent bacteria detected in AE-COPD patients

Bacterial species	Respiratory failure		Group of COPD		Stage of COPD				Severity of AE-COPD	
	Yes	No	B	D	I	II	III	IV	Non-severe	Severe
*K. pneumoniae*	44 (60.27)	33 (50.77)	9 (69.23)	68 (54.40)	5 (55.56)	27 (58.70)	39 (54.17)	6 (54.55)	44 (63.77)	33 (47.83)
*H. influenzae*	16 (21.92)^*^	6 (9.23)^*^	2 (15.38)	20 (16.00)	0 (0.00)	10 (21.74)	11 (15.28)	1 (9.09)	9 (13.04)	13 (18.84)
*M. catarrhalis*	6 (8.22)	11 (16.92)	1 (7.69)	16 (12.80)	2 (22.22)	3 (6.52)	10 (13.89)	2 (18.18)	5 (7.25)	12 (17.39)
*S. pneumoniae*	13 (17.81)	10 (15.38)	2 (15.38)	21 (16.80)	4 (44.44)^†^	8 (17.39)^†^	7 (9.72)^†^	4 (36.36)^†^	10 (14.49)	13 (18.84)
**Total**	**73**	**65**	**13**	**125**	**9**	**46**	**72**	**11**	**69**	**69**

The values are given as numbers (%)

^*^p = 0.042 and ^†^*p* = 0.014 between these groups

AE-COPD, acute exacerbation of chronic obstructive pulmonary disease

### CRP level as a marker of pneumonia in AE-COPD patients

**Fig. 2:**
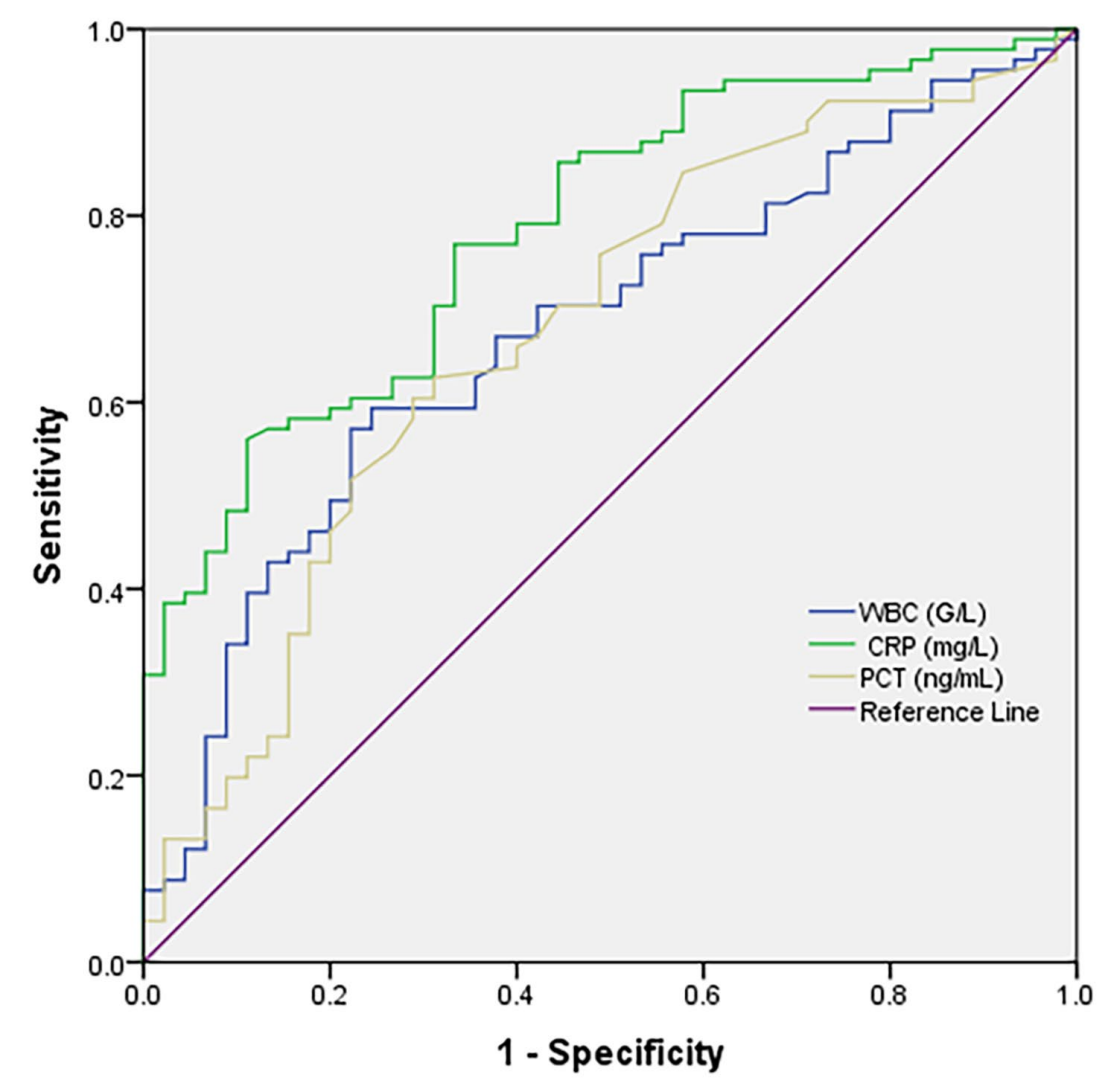
Diagnostic performance of several biomarkers for distinguishing between AE-COPD patients with and without pneumonia. WBC, white blood cell; CRP, C-reactive protein; PCT, procalcitonin

To evaluate whether certain biomarkers can differentiate between AE-COPD patients with and without pneumonia, we performed a receiver operating characteristic (ROC) curve analysis (Fig. 2). CRP, PCT, and WBC count were utilized to identify pneumonia patients. The findings revealed that the CRP concentration exhibited substantial discriminatory power for distinguishing between pneumonia patients and controls (AUC = 0.78, *p* < 0.001). Conversely, the discriminative ability of PCT and WBC count was comparatively weaker, with AUC values of 0.66 and 0.67 (*p* = 0.001), respectively. The calculated cutoff point for CRP was determined to be 40.8 mg/dL, achieving a sensitivity of 56% and specificity of 87% in diagnosing pneumonia.

## Discussion

To the best of our knowledge, this study represents the first investigation on pathogens among AE-COPD patients in Vietnam, with a comparison between those with and without pneumonia. The relationship between AE-COPD and pneumonia is indeed intricate; pneumonia is sometimes perceived as a potential cause of AE-COPD [27, 28], while others view it as a distinct clinical and pathological entity [29, 30]. Moreover, pneumonia tends to be underdiagnosed in COPD patients. This misdiagnosis could lead to the inappropriate use of inhaled corticosteroids and consequently result in worsened outcomes [10, 11]. Hence, our research significantly contributes to this area. We detected infections in 92.39% and 86.96% of patients with pneumonia and controls, respectively. Infection rates in AE-COPD patients vary across different countries. For instance, in Korea, 55.3% of cases were affected [31], while in China, bacterial infections accounted for 34.28% [15]. In the UK, among admissions where a sputum sample was collected, 24.6% had bacterial growth, and 46.7% tested positive for viral infections [32]. Additionally, a year-long prospective epidemiological study conducted in the Asia-Pacific region, including Hong Kong, the Philippines, South Korea, and Taiwan, revealed that during AE-COPD visits, bacterial pathogens were prevalent in 78.0% and 74.7% of sputum samples analyzed via culture and real-time PCR, respectively [33]. It seems possible that our relatively higher rates are due to regional variation and the application of dual techniques.

 The present study identified *K. pneumoniae, H. influenzae*, and *M. catarrhalis* as the most common bacteria detected, followed by *S. pneumoniae and S. mitis* in both groups. These results suggest that Gram-negative bacteria may be the predominant cause of AE-COPD in Vietnam. These align with studies conducted in China [15], the Philippines [33], South Korea [33], and Taiwan [33, 34]. However, these findings differ significantly from those in Hong Kong [33] and Western countries such as the UK [32], where community-acquired pneumonia (CAP) (*H. influenzae, S. pneumoniae, and M. catarrhalis*) was associated with AE-COPD. Interestingly, these outcomes are contrary to those of Mood et al. in India [35] and Mussema et al. in Ethiopia [36]. These authors found that the most common bacterial isolate was *P. aeruginosa*, followed by *K. pneumoniae* and either *S. pneumoniae* or *S. aureus*. Viruses also play a significant role in emergency admissions for COPD. A multicenter study conducted in South Korea revealed that viruses were identified in 33.2% of AE-COPD patients, with 10.9% showing bacterial and viral coinfections [31]. Other reports also showed a notably high percentage of positive virus samples (46.7%) in the UK [32] and 35.6% in the Asia-Pacific region [33]. Almost all the studies reported influenza as the most frequently isolated virus, followed by rhinovirus and respiratory syncytial virus. Consistent with the literature, this research also revealed influenza virus to be the most prevalent among the detected viruses.

 In our study, real-time PCR identified seven typical and three atypical bacteria. Notably, *K. pneumoniae, H. influenzae, and S. pneumoniae* were more frequently observed by real-time PCR than by conventional sputum culture. This difference could be attributed to the potential reduction in bacterial numbers due to prior antibiotic use in some patients. Conversely, *M. catarrhalis, A. baumannii*, and *P. aeruginosa* were frequently detected by conventional sputum culture. In accordance with previous reports [33, 37], the current study has supported the significant enhancement of identifying the infectious cause of AE-COPD through the combination of real-time PCR with conventional methods, particularly by increasing the detection of atypical bacteria and viruses. Although microbiological culture has provided valuable insights into the sputum microbial composition in AE-COPD patients, identifying its inherent limitations is crucial. Challenges such as false negative results, cultivation biases, viability requirements, time-consuming procedures, and limited microbial diversity hinder the identification of specific pathogens and the acquisition of a comprehensive microbial profile [38]. Acknowledging these limitations, research advocates for increased integration of advanced molecular techniques, such as real-time PCR, to increase the sensitivity and detect specific DNA sequences related to pathogenic species. This could contribute significantly to its clinical diagnostic value [39].

The four common bacterial species isolated in the present study were opportunistic pathogens [15, 32–34, 37], which are known to cause a long-term disease course in AE-COPD patients. These bacteria have been observed to exhibit significantly varied antibiotic resistance profiles. In the control group, *K. pneumoniae* remained sensitive to almost all β-lactams tested, except for ampicillin. These findings align with those of Ma et al. (2015) [15] and Mussema et al. (2022) [36]. In contrast, *K. pneumoniae* showed resistance to this antibiotic group, including carbapenem among AE-COPD patients with pneumonia. This raises the possibility of different antimicrobial resistance mechanisms occurring in this microorganism, possibly due to β-lactamase production or modification of the antibiotic target through genetic mutations or post-translational modification [40]. In this study, *M. catarrhalis* demonstrated the highest resistance to levofloxacin and ciprofloxacin, followed by sulfamethoxazole/trimethoprim, in both groups. This finding is quite different from those of several previous publications. Feng et al. (2013) reported that both β-lactams and fluoroquinolones were competently effective against this bacterium [41]. However, Smith et al. (2021) highlighted the high degree to which *M. catarrhalis* is resistant to β-lactam antibiotics [42]. According to the literature, *H. influenzae*, a fastidious bacterium, has specific and intricate growth requirements, posing challenges for conventional antibiograms in routine laboratory settings [43]. Additionally, *S. pneumoniae* exhibits various serotypes with distinct antibiotic susceptibilities, making it difficult to create universally applicable antibiograms [44]. These complexities are key factors limiting the study of antibiogram tests for these bacteria. Therefore, instead of focusing on *H. influenzae* and *S. pneumoniae*, our analysis included *A. baumannii* and *S. mitis*. While *A. baumannii* is a significant pathogen in nosocomial infections, community-acquired Acinetobacter infection is becoming increasingly important. This is due to antibiotic resistance and its impacts on patients with underlying comorbidities [45]. Indeed, our study, as well as previous reports [15, 41], noted that it was resistant to almost all tested antibiotics. Imipenem and meropenem remained the most powerful antimicrobial agents against *A. baumannii*, even though the resistance rates reached 43%. In case of carbapenem-resistant *A. baumannii*, a long course of colistin therapy serves as an alternative approach [46]. The outcomes of the antibiogram test for *S. mitis* between the two groups varied significantly in this study, leading to interesting findings (Fig. 1D). *S. mitis* is one of the normal flora in the oral tract and is the main pathogen of infective endocarditis. However, a significant increase in the incidence of *S. mitis* was noted in patients with the asthma-COPD overlap (ACO) phenotype [47]. Notably, its in vitro antimicrobial properties against respiratory pathogens have been determined [48], which could explain this observation.

As mentioned in the literature review [7], bacterial infections significantly worsen COPD exacerbations, and the prevalence of pathogens varies globally. Hence, our third research question delved into potential associations between clinical characteristics and common pathogenic etiologies in AE-COPD patients. Despite previous studies highlighting *H. influenzae* as a primary pathogen in acute exacerbations [7, 49], a comprehensive analysis of its role in pathogen-related characteristics has not been performed. The results we observed contribute to the existing database and stimulate further interest and consideration in this area. One study evaluated the clinical features and prognostic indicators of lower respiratory tract infections caused by *H. influenzae* and revealed a respiratory failure rate of up to 56% [50]. Furthermore, another study elucidated a distinct genetic profile in *H. influenzae* strains associated with exacerbations compared to those associated with asymptomatic chronic infection [51]. These findings suggest that complex interactions between host immunity and the pathogenicity of the infecting strain of *H. influenzae* may play a crucial role in determining the outcome of infection. Our data also revealed significant differences in the prevalence of *S. pneumoniae* among patients with various stages of COPD, with the highest incidence observed among patients with stage I and IV COPD. This finding is consistent with a previous report [52], which showed that *S. pneumoniae* is frequently present in early- and late-stage patients compared to those in the middle stage. The relationship between sputum bacteriology and COPD severity remains unclear. Factors reported to influence sputum bacteriology in AE-COPD include patient characteristics, antibiotic pre-treatment, current smoking status, duration between exacerbations, compliance with inhalation medication, and influenza vaccination [52]. Lin et al. [34] reported a greater incidence of *K. pneumonia* infection in patients with stage I COPD than in those with stage II, III, or IV COPD, while Ko et al. [53] highlighted a higher incidence of *H. influenzae* infection in patients with better lung function. Combined with our results, it is possible that the structural lung changes leading to the alteration in FEV_1_ are associated with the higher rate of bacterial load in the airway. These findings underscore the complexity of the bacterial distribution in AE-COPD patients and emphasize the need for a comprehensive investigation into the underlying mechanisms involved.

Another significant discovery in this study involved identifying indicators to distinguish between AE-COPD cases with and without pneumonia. The analysis of ROC curves revealed that, among the biomarkers evaluated, CRP was the most effective discriminator. These findings are consistent with the literature, which highlights CRP as a valuable biomarker for inflammation and infection, including pneumonia [54]. CRP is also known as an independent indicator of severity in patients with CAP [55]. Flanders et al. demonstrated that in adults presenting with acute cough, a CRP level ≥ 40 mg/L exhibited a sensitivity of 70% and a specificity of 90% in distinguishing between those with radiographically confirmed CAP and others [56]. Similarly, another study utilized a CRP value of ≥ 40 mg/L as a secondary diagnostic criterion for pneumonia [57]. In the present study, although the CRP concentration exhibited good specificity (87%), its sensitivity was relatively modest (56%). This finding suggested that relying solely on CRP levels may lead to missed pneumonia diagnoses in certain patients. The inconsistency may be due to the underlying mechanism of COPD, which is characterized by chronic inflammation. Therefore, clinical examination and consideration of other diagnostic factors remain essential.

There are several limitations to this study. The lack of microbiological data from various methods, such as serological tests, urine antigen assays, or nasopharyngeal lavage immunochromatography or immunofluorescence assays, may result in a less comprehensive analysis [8]. However, respiratory samples continue to be the primary choice for pathogen detection in AE-COPD patients. Furthermore, employing dual techniques in our study could enhance detection capabilities. Due to the small sample size of positive culture species, the current study did not allow for statistical analysis of antibiotic resistance between these two groups, however, it partially revealed differences between AE-COPD patients with and without pneumonia. Additional research is also needed to better understand the treatment outcomes of these groups. Nevertheless, our findings still hold potential utility in clinical practice.

## Conclusion

This study provides comprehensive insight into the distribution of pathogens and antibiotic susceptibility and their associations with clinical characteristics among AE-COPD patients, both with and without pneumonia. These results could lead to valuable insights for developing effective treatment approaches, underscoring the importance of considering bacterial diversity and antibiotic responses during AE-COPD management. The efficacy of empirical antibiotic therapy also becomes a significant challenge. Hence, it is imperative for physicians and pharmacists worldwide to recognize the urgent need for rigorous surveillance and infection control strategies in combating the growing bacterial resistance. Continuous monitoring of bacterial sensitivity and resistance patterns is essential. Additionally, the study emphasizes the ongoing need for advancements in diagnostic methods.

### Electronic supplementary material

Below is the link to the electronic supplementary material.

Supplementary Material 1

## Data Availability

All data supporting the findings of this study are available within the paper and its Supplementary Information.

## Abbreviations

AE-COPD: Acute Exacerbations of Chronic Obstructive Pulmonary Disease
CAP: Community-acquired pneumonia
CRP: C-reactive protein
PCT: Procalcitonin
