# Supplementary material for: Spectrum and antimicrobial resistance in acute exacerbation of chronic obstructive pulmonary disease with pneumonia: a cross-sectional prospective study from Vietnam
Source: BMC Infect Dis. 2024 Jun 23;24:622. doi: 10.1186/s12879-024-09515-6 (PMC11194910; doi:10.1186/s12879-024-09515-6)
Supplement: Supplementary file 1 — Supplementary Material 1 [file 12879_2024_9515_MOESM1_ESM.docx]

**Supplementary information**

**Title:** Spectrum and antimicrobial resistance in acute exacerbation of chronic obstructive pulmonary disease with pneumonia: A cross-sectional prospective study from Vietnam

**Authors:** Duy Tuyen Dao^1†^, Huu Y Le^2,3†^, Huu Song Le^3,4*^, Minh Hai Nguyen^1,3^, Thi Duyen Thi^1,3^, Xuan Dung Nguyen^1^, Thanh Thuyet Bui^4,5^, Thi Huyen Trang Tran^4,6^, Van Luan Pham^1,3^, Hang Nga Do^2^, Jim-Tong Horng^2,7,8*^, Dinh Tien Nguyen^1,3*^

**Table S1. Blood culture result in AE-COPD with and without pneumonia.**

| **Pathogens** | **AE-COPD with pneumonia**  **(N = 92)** | **AE-COPD only**  **(N = 46)** |
| --- | --- | --- |
| **Identified results** | | |
| Negative | 91 (98.9) | 45 (97.8) |
| Positive | 1 (1.1) | 1 (2.2) |
| **Pathogen detected subgroup** | | |
| Bacterial species | *Sphingomonas paucimobilis* | *Escherichia coli* |

Values are given as number (%).

AE-COPD, acute exacerbation of chronic obstructive pulmonary disease.

**Table S2.** Antibiotic profile of *H. influenzae* in two positive patients.

| **Antibiotic** | **Patient 1** | **Patient 2** |
| --- | --- | --- |
| Sulfamethoxazole/Trimethoprim | R | R |
| Rifampicin | S | S |
| Ertapenem | S | S |
| Ciprofloxacin | S | S |
| Meropenem | S | S |
| Cloramphenicol | S | R |
| Azithromycin | S | S |
| Cefotaxime | S | S |
| Cefuroxime | S | S |
| Ampicillin | R | R |
| Ampicillin/Sulbactam | S | S |
| Amoxicillin/clavulanic acid | S | I |

S, sensitive; I, intermediate; R, resistant.

**Table S3.** Antibiotic profile of *S. pneumoniae* in two positive patients.

| **Antibiotic** | **Patient 1** | **Patient 2** |
| --- | --- | --- |
| Sulfamethoxazole/Trimethoprim | R | R |
| Levofloxacin | S | S |
| Moxifloxacin | S | S |
| Linezonid | S | S |
| Rifampicin | S | S |
| Erythromycin | R | R |
| Ertapenem | R | R |
| Clindamycin | R | R |
| Imipenem | R | R |
| Tetracyclin | R | R |
| Vancomycin | S | S |
| Tigercycline | S | S |
| Cefotaxime | R | R |
| Cefuroxime | R | R |
| Cloramphenicol | S | R |
| Doxycycline | R | R |

S, sensitive; R, resistant.
